# The effect of ocean warming on black sea bass (*Centropristis striata*) aerobic scope and hypoxia tolerance

**DOI:** 10.1371/journal.pone.0218390

**Published:** 2019-06-13

**Authors:** Emily Slesinger, Alyssa Andres, Rachael Young, Brad Seibel, Vincent Saba, Beth Phelan, John Rosendale, Daniel Wieczorek, Grace Saba

**Affiliations:** 1 Center for Ocean Observing Leadership, Department of Marine and Coastal Sciences, School of Environmental and Biological Sciences, Rutgers University, New Brunswick, NJ, United States of America; 2 College of Marine Science, University of South Florida, St. Petersburg, FL, United States of America; 3 National Oceanic and Atmospheric Administration (NOAA), Northeast Fisheries Science Center, Geophysical Fluid Dynamics Laboratory, Princeton, NJ, United States of America; 4 National Oceanic and Atmospheric Administration (NOAA), Northeast Fisheries Science Center, James J. Howard Laboratory, Highlands, NJ, United States of America; University of Connecticut, UNITED STATES

## Abstract

Over the last decade, ocean temperature on the U.S. Northeast Continental Shelf (U.S. NES) has warmed faster than the global average and is associated with observed distribution changes of the northern stock of black sea bass (*Centropristis striata*). Mechanistic models based on physiological responses to environmental conditions can improve future habitat suitability projections. We measured maximum, standard metabolic rate, and hypoxia tolerance (S_crit_) of the northern adult black sea bass stock to assess performance across the known temperature range of the species. Two methods, chase and swim-flume, were employed to obtain maximum metabolic rate to examine whether the methods varied, and if so, the impact on absolute aerobic scope. A subset of individuals was held at 30°C for one month (30_chronic_°C) prior to experiments to test acclimation potential. Absolute aerobic scope (maximum–standard metabolic rate) reached a maximum of 367.21 mgO_2_ kg^-1^ hr^-1^ at 24.4°C while S_crit_ continued to increase in proportion to standard metabolic rate up to 30°C. The 30_chronic_°C group exhibited a significantly lower maximum metabolic rate and absolute aerobic scope in relation to the short-term acclimated group, but standard metabolic rate or S_crit_ were not affected. This suggests a decline in performance of oxygen demand processes (e.g. muscle contraction) beyond 24°C despite maintenance of oxygen supply. The Metabolic Index, calculated from S_crit_ as an estimate of potential aerobic scope, closely matched the measured factorial aerobic scope (maximum / standard metabolic rate) and declined with increasing temperature to a minimum below 3. This may represent a critical threshold value for the species. With temperatures on the U.S. NES projected to increase above 24°C in the next 80-years in the southern portion of the northern stock’s range, it is likely black sea bass range will continue to shift poleward as the ocean continues to warm.

## Introduction

Marine environments are progressively warming as a consequence of climate change [1]. Along the U.S. Northeast Shelf (U.S. NES), annual ocean temperature is rising faster than the global average [2,3] resulting in rapid temperature increases [4,5] with a strong warming signal prominent during spring, summer and fall [4]. Over the next 80-years, sea surface and bottom temperatures on the U.S. NES are projected to rise an additional 4.1°C and 5.0°C, respectively [6,7]. Contemporary ocean warming on the U.S. NES has been associated with distribution shifts of many economically and ecologically important fish species both in latitude and/or depth [7–11], associated with tracking local climate velocities [12]. Understanding and projecting shifts in fish distribution will be important for characterizing potential ecological and economic impacts and anticipating and resolving fishery management conflicts [13].

Temperature directly affects metabolic rates of marine ectotherms [14,15] and is believed to set the boundaries of species ranges [16–18]. One explanation for the effects of temperature on ectothermic species’ physiology is the oxygen and capacity-limited thermal tolerance hypothesis (OCLTT; [19,20]) that postulates thermal limitation occurs due to a mismatch in oxygen demand and supply at sub-optimal temperatures, and can ultimately determine metabolically suitable habitat [21]. In this framework, the thermal optimum occurs where absolute aerobic scope (AAS), the difference between maximum (MMR) and standard metabolic rate (SMR) [22], is highest. SMR is the cost of maintenance for an organism and increases exponentially with temperature [15]. MMR initially increases with temperature but may respond differently at high temperatures due to impaired oxygen supply or utilization. Importantly, MMR measurements can vary substantially depending on the method employed, typically an exhaustive chase or a swim-flume method [23,24]. Varying MMR measurements can affect the AAS measurement, and thus the interpretation of temperature effects on AAS. The decrease in AAS beyond the thermal optimum is associated with the differing thermal sensitivities of MMR and SMR [25] and suggests that these temperatures are suboptimal. AAS is thought to represent the capacity for oxygen uptake, beyond what supports maintenance metabolism, that can be utilized for activities that promote individual fitness (e.g. growth, reproduction, predator avoidance; [26]). However, it should be noted that there are exemptions to this hypothesis found in other fish species [27–29], including our own experiments, and this discrepancy is further discussed in Jutfelt et al. [30]. Nonetheless, the adaptive benefit of living at suitable temperatures to maintain aerobic scope may provide a mechanistic explanation for fish distribution patterns.

The general distribution of fishes is broadly confined by thermal preferences. Oxygen availability can further constrain metabolically suitable habitat within thermal boundaries because environmental oxygen solubility decreases with warmer temperatures ([31], but see [32]). Simultaneously, warmer temperatures can increase fish oxygen demand [33,34], which can potentially reduce hypoxia tolerance from both a decrease in oxygen solubility and an increase in fish oxygen demand [35,36]. The hypoxia tolerance of a fish can be estimated as the critical oxygen saturation level (S_crit_), which is the % O_2_ air-saturation (%O_2_) below which oxygen supply cannot match the demands of maintenance metabolism. Further reductions in %O_2_ cause a proportional decrease in SMR [37]. Below the S_crit_, ATP production relies on unsustainable anaerobic pathways that can lead to a host of biochemical challenges including a buildup of anaerobic end products and acid-base chemistry changes [38,39], contributing to time-limited survival if the fish remains in water with oxygen levels below S_crit_. Generally, a fish with a low S_crit_ is thought to be more tolerant of lower sustained oxygen levels [40]. Additionally, the S_crit_ further provides a means of calibrating the Metabolic Index (MI). Deutsch et al. [17] proposed the MI as the ratio of environmental oxygen supply to animal oxygen demand, which is effectively an estimate of a species’ time-averaged factorial aerobic scope. By definition, the MI is equal to 1 when the environmental %O_2_ is equal to S_crit_. The MI also contains a temperature dependency term (E_o_), which is calibrated by the S_crit_ at a given temperature, and takes into account temperature effects on the ratio of oxygen supply to fish demand. A minimum MI of 2–5 indicates the environmental capacity to supply oxygen at 2-5x the rate required to support metabolic needs at rest and is considered supportive of a population. This has delineated the equatorward distribution limit for a diverse group of marine fishes and invertebrates (reviewed in [17]). The measurement of S_crit_ and subsequent calculation of a thermally sensitive MI have proven useful in predicting habitat suitability.

**Fig 1:**
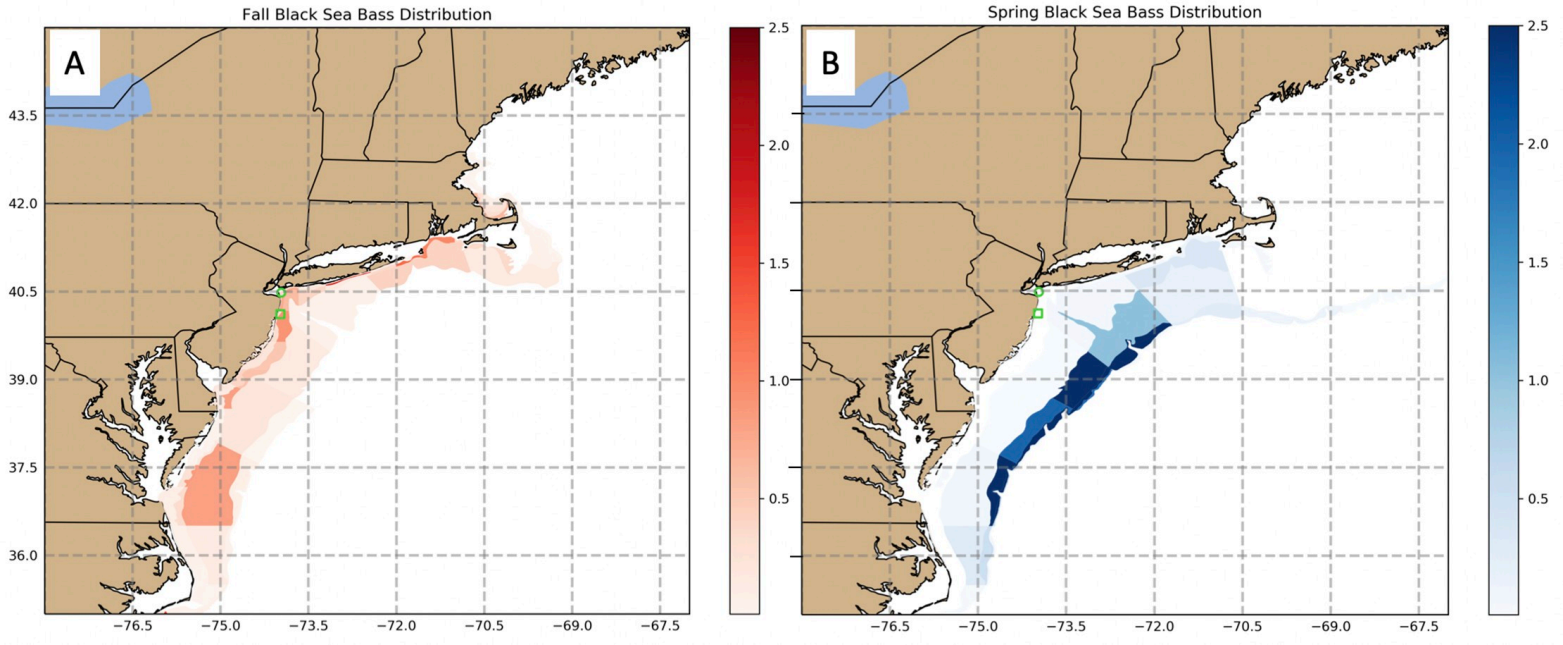
Seasonal black sea bass distribution throughout their range on the U.S. Northeast Shelf. Black sea bass distribution is shown as mean strata CPUE (1980–2017) from the NOAA NMFS (National Marine Fisheries Service) bottom trawl survey (https://www.nefsc.noaa.gov/femad/ecosurvey/mainpage/). The color bars represent mean CPUE (kg tow^-1^) where darker shades indicate a higher average CPUE. NMFS survey fall, typically September-November (A; red) and spring, typically February-April (B; blue), black sea bass distribution shows black sea bass inshore and offshore habitat, respectively. Green square = collection site in 2016; green circle = collection site in 2017.

The northern stock of black sea bass (*Centropristis striata*) on the U.S. NES extends from Cape Hatteras to the Gulf of Maine and is centered in the Mid-Atlantic Bight (MAB; [41]). These fish seasonally migrate from the continental shelf edge in cooler months to inshore depths (5-50m) in warmer months ([42,43]; Fig 1). Seasonally migrating black sea bass thus experience a wide range of temperatures throughout the year, ranging from 6°C during winter and up to 27°C during summer/early fall months [44]. While these are average seasonal temperatures, this region experiences large interannual variations in surface and bottom temperatures [45,46]. In addition, under a predicted doubling of anthropogenic atmospheric CO_2_ in the next 80 years, summer bottom temperature could reach 30°C in the southern portion of black sea bass range [6]. This could potentially limit the southern inshore extent of black sea bass habitat. Off the coast of New Jersey, periodic hypoxic events (e.g. O_2_ concentration < 2.2 mg L^-1^ at 14°C) can occur during the summer as a result of high biological activity [47] fueled by upwelling of nutrient rich waters [48]. Therefore, during the warm summer months oxygen limitation in hypoxic regions along the U.S. NES may also reduce metabolically available habitat for black sea bass.

The northern stock of black sea bass may already be exhibiting poleward shifts, likely due to ocean warming [7,49]. Evidence for current black sea bass distribution shifts comes primarily from bottom trawl survey data [7]. Laboratory-based process studies focused on the physiology of an organism provide detailed mechanistic relationships between the environment and the animal [50]. Results from these physiological studies are useful for modeling metabolically suitable habitat based on environmental parameters [51] and could be used to model current black sea bass distributions (e.g., [17,52]) and project future distribution shifts with continued ocean warming.

There were three aims for this study. First, we measured AAS and S_crit_ (for MI calculation) for the northern stock of adult black sea bass over a range of temperatures experienced inshore to compare, if present, thermal optima. These parameters can potentially be used in future habitat suitability modeling and assessing future shifts in black sea bass distribution. Second, we tested the ability of black sea bass to acclimate to an extreme warm temperature (30°C) given the high likelihood such temperatures will become increasingly common under future climate change projections. A subset of black sea bass were acclimated to 30°C for one month and their aerobic performance was compared to those fish tested under short-term acclimations. And finally, we compared two different MMR measurement methods, chase and swim-flume methods, to investigate which method performed better for black sea bass.

## Materials and methods

### Fish collection and husbandry

Adult black sea bass (*Centropristis striata*) from the northern stock (length = 221-398mm; weight = 193.7–700.4g) were collected off the coast of New Jersey, USA at depths of 15-20m in early June from Sea Girt Reef (40°7’07”N, 73°58’42”W) by fish traps (June 14–21 2016), and from local reefs off Sandy Hook (40°28’46”N, 73°57’47”W) by hook-and-line (June 28 –July 5 2017). Once captured, fish were housed in the NOAA James J. Howard Marine Laboratory, held at ambient temperature (22 ± 1°C) and salinity (26ppt), maintained at a natural photoperiod for New Jersey summer (14h:10h light:dark), and fed daily to satiation on a diet of sand lance and silversides for the duration of the respirometry experiments. Water temperature and salinity was monitored daily using a YSI (Pro-30; Yellow Springs, Ohio, USA), and water chemistry remained at suitable levels (< 20 uM nitrate, undetectable nitrite, < 0.05 uM ammonia, pH range of 7.98–8.04). Fish were acclimated to captive conditions for a minimum of two weeks prior to the trials, after which all experimental fish ate regularly and were in good condition. The time from fish collection to experiment trials ranged from two to four weeks. After acclimation, fish were measured for length (TL mm), weight (g), and tagged with individually numbered T-bar Floy tags inserted underneath the dorsal rays. For each temperature treatment, fish were acclimated at a rate of 2°C day^-1^ to reach experimental temperature, then held at the target treatment temperature for at least 48hr prior to the start of experiments. We defined this, and refer to this process throughout, as a short-term acclimation. Fish were starved 48hr prior to the start of each experiment to eliminate effects of specific dynamic action [53]. A total of 152 experimental fish were used during 2016 and 2017 (S1 Dataset).

### Experimental setup

Experimental tanks (1,200L) were filled with treated seawater from Sandy Hook Bay that continuously circulated through a closed system. Circulating seawater was treated using filters (sand and biological) and UV-light, and salinity was adjusted to mimic average summertime inshore NJ bottom water (32±1 ppt). Experimental temperatures were achieved using in-line chillers (Aqua Logic Delta Star; San Diego, California, USA) and/or titanium exchanger heaters (Innovative Heat Concepts, Homestead, Florida, USA), and maintained at ±1°C from target temperature.

Metabolic rates were measured using intermittent respirometry under the protocols outlined in Clark et al. [54] and Svendsen et al. [55]. Flow-through respirometers (13.5L; 23[H]x26[W]x37[L] cm plexiglass) were placed into the two experimental tanks (two respirometers per tank; four respirometers per trial). Flush pumps (Eheim Universal 600 l//h; Deizisau, Germany) connected to the respirometer were used to pull water from the surrounding temperature bath to replenish dissolved oxygen and eliminate metabolic waste buildup within the respirometer. The duration and timing of flushes set the intermittent cycles, which were controlled through a pre-determined time sequence using a DAQ-M instrument (Loligo Systems; Viborg, Denmark), and were determined based on the trial temperature so that % O_2_ air-saturation (%O_2_) remained above below 75% [56]. See S1 Table for list of intermittent flushes at each temperature. For each closed measure period (when flush pumps were off), the rate of decline in dissolved oxygen concentration within the sealed respirometer was used to calculate a mass specific rate of oxygen consumption, a proxy for metabolic rate. A closed recirculation loop connected with a smaller pump (Eheim Universal 300 l/h; Deizisau, Germany) was also utilized to uniformly disperse dissolved oxygen within the respirometer and provide waterflow across the oxygen dipping probe optical mini sensor (PreSens Pst3; Regensburg, Germany). Oxygen probes were calibrated in accordance with the supplier’s manual (Oxygen dipping probe PSt3, PreSens GmbH, Regensburg, Germany) and checked with a YSI (ProSolo ODO; Yellow Springs, Ohio, USA) that was calibrated in 100 and 0%O_2_ sample waters. Autoresp computer software (Loligo Systems; Viborg, Denmark) and a Witrox-4 instrument (Loligo Systems; Viborg, Denmark) were used to continuously monitor dissolved oxygen and temperature within the respirometer over the course of the experiment.

Intermittent respirometry was also used in hypoxia experiments to control for CO_2_ and metabolite build up within the respirometer [57]. In this set up, each respirometer flush pump was connected to a separate external water reservoir containing the same system water. Within the external water reservoir, a pump (Eheim Universal 1200 l/h; Deizisau, Germany) was utilized to provide uniform mixing and to provide flow across an oxygen optode to monitor source %O_2_ and served as a mixing device. Four small microdiffusers were connected to a N_2_ gas canister [37] and utilized for diffusion of nitrogen gas, and a subsequent displacement of O_2_, within the external bath. N_2_ was manually released using a nitrogen purge regulator (Randor SR5B-580 Airgas; Paris, France) allowing for monitoring of PSI within the canister and being released into the external water reservoir. For fine scale tuning of the %O_2_ in the external water bath, system water was periodically pumped into the external reservoir and was used to replenish water supply. All changes in the %O_2_ levels were performed during a closed measure period when the experimental respirometers were closed to external water flow to avoid fluctuations in the %O_2_ level within the individual respirometers.

Experiments were conducted at a range of temperatures (12, 17, 22, 24, 27 and 30°C). For both 2016 and 2017 experiments, we conducted a short-term acclimation on black sea bass before the start of each aerobic scope trial (*see Fish Collection and Husbandry)*. In addition to short-term acclimation trials at each temperature, we also included a temperature treatment with chronic exposure (one month) to 30°C (30_chronic_°C) because projections of ocean warming within the next century predict summer bottom temperatures as high as 30°C in the southern portion of black sea bass range [6]. This allowed for testing of current black sea bass acclimation potential by assessing the effects of long-term exposure to 30°C on aerobic scope and hypoxia tolerance. Sample sizes for all temperature treatments are in the S1 Dataset.

We used two different methods in an attempt to elicit maximum metabolic rate (MMR): exhaustive-chase and swim-flume. MMR was tested using two different methods as the method used can affect the resulting metabolic rates and thus AAS [23–24,58]. Therefore, a comparison of AAS resulting from the “chase” and “swim-flume” methods was conducted. For the chase method, individual black sea bass were placed in a 4ft-diameter chase tank filled with water from the experimental tanks. Fish were chased to exhaustion via tactile stimulation on the caudal fin. Exhaustion was determined as the point where fish became unresponsive to further tactile stimulation and air exposure. Fish were then immediately transferred and sealed within individual respirometers within ~1 minute from the end of the chase. Fish remained within respirometers for ~23hr allowing for recovery and subsequent standard metabolic rate (SMR) measurement [59]. Sixteen fish were used in each temperature treatment. In the 30_chronic_°C temperature treatment, the sample size was nine fish due to the removal of five fish in poor condition before and two fish during experiments. At the end of SMR measurements, the first twelve fish rested for at least 24hr and then exercised in a swim-flume. The last four fish of each temperature treatment remained in the respirometer for hypoxia testing (see below). For the swim-flume, individual fish were exercised in an acrylic Brett-style flume respirometer (Loligo Systems 90L; Viborg, Denmark). Fish were placed within the working section of the flume (20[H] x 20[W] x 70[L] cm) and sealed within the flume for the duration of the experiment. A motor driven propeller, housed within the flume and separate from the working section, was utilized for both manual speed changes to allow for measurements across different activity levels and for continuous uniform mixing of water (and O_2_) throughout the chamber. The flume was fully submerged within an external water bath (71[H] x 35[W] x 188[L] cm), to maintain consistent temperature throughout the trial. A pump (Eheim Universal 1200 l/h; Deizisau, Germany) was utilized for intermittent flushing of new system water into the flume chamber after measure periods and to supply system water to the external bath during measure periods. Fish were exercised using a sprint protocol. First, the fish was allowed to adjust to the swim flume for 10 minutes with minimal flow to provide mixing. Then, the flow was slowly increased to a swimming speed of 0.95 BL s^-1^, the lowest speed black sea bass began to swim, over a five-minute period. The fish adjusted to this speed for ~10 minutes. After the adjustment period, the speed was incrementally increased over a period of five minutes until the fish was sprinting (designated as >10 bursts utilizing the caudal fin during 30s intervals and an inability to maintain position in the working section without burst swimming). Once the fish reached their sprinting speed, the flush pump was turned off and the flume was sealed to allow measurement of metabolic rate. Fish were held at their sprint speed for a period of 10 minutes or until failure, determined when the fish rested at the backgrate for >10s.

Background respiration was measured by taking background MO_2_ (MO_2br_) pre- and post-trial in empty respirometers for ~1.5hr. A linear regression between pre- and post-MO_2br_ was used to apply a correction factor to each MO_2_ value recorded throughout an experiment.

### Critical %O_2_ determinations

Hypoxia (S_crit_) experiments were conducted on the last four fish of each temperature treatment trial. This allowed for reliable use of fish that were already acclimated to the respirometers and had reached SMR overnight. S_crit_ was measured by incrementally decreasing the %O_2_ in the respirometers [37]. We measured ~10%O_2_ bins. The number of bins was temperature dependent based on where S_crit_ occurred. The experiment started at 100%O_2_ and incrementally decreased by 10%O_2_ (100, 90, 80, 70%, etc.) until S_crit_ was reliably reached, indicated by a significant decrease in metabolic rate and deviation in SMR. Consequently, as hypoxia tolerance decreased at higher temperatures, the number of %O_2_ bins in the experiment decreased as well. At each %O_2_ bin there were three intermittent (flush, wait, measure) cycles measured which collectively took ~30 minutes depending on the temperature. If a fish lost equilibrium or exhibited signs of distress, the experiment was immediately ended for that individual.

### Ethics statement

Husbandry and experiments were conducted according to relevant national and international guidelines. Fish were collected under permits #1610 & #1717 issued by the New Jersey Department of Environmental Protection. No endangered or protected species were involved. Protocols for the treatment and euthanasia procedure of all animals reported here was approved by the Rutgers University Institutional Animal Care and Use Committee protocol number 15–054. All efforts were made to ensure minimal pain and suffering. Fish behavior, feeding, and condition were monitored daily. Any fish exhibiting apparent health issues or excessive stress (i.e. lack of appetite, difficulties with buoyancy or orientation) were not used in experiments. Fish that could not recover apparent health issues and exhibited extreme distress were euthanized with an overdose of MS-222 (250 mgL^-1^). Between 2016 and 2017, 152 of 164 fish were used for experiments. Ten fish were in poor condition and two fish continued to experience symptoms of barotrauma (i.e. exophthalmia) prior to experiments and were not used. Three fish showed signs of distress during an experiment and were immediately removed and monitored. When condition did not improve, the fish were euthanized. All of these mortalities were associated with the 30_chronic_°C and 30°C temperature treatments. All experimental animals were euthanized at the end of the experiment with MS-222 (250 mgL^-1^) to obtain a final sex of the fish and to prevent any potential spread of pathogens or infectious disease to natural populations that may have resulted from prolonged captivity in the laboratory (~2 months) and gone undetected.

### Data analysis

Fish MO_2_ is presented as mass-specific (MO_2_: mgO_2_ kg^-1^ hr^-1^) and was calculated from the slope of oxygen saturation decline during each closed measure period using the equation:

$$MO_{2}=([O_{2}{]}_{t0}-[O_{2}{]}_{t1})∙\frac{V}{t}∙\frac{1}{BW}$$

where MO_2_ is mass-specific metabolic rate (in mgO_2_ kg^-1^ hr^-1^), [O_2_]_t0_ is oxygen concentration (mgO_2_/L) at time t = 0, [O_2_]_t1_ is the oxygen concentration at time t = 1, V is the respirometer volume (L) without the fish volume, t is t_1_-t_0_ (hr) referring to one measure period, and BW is the body weight (kg) of the fish. The MO_2_ was automatically calculated after each measurement period in the AutoResp program during the experiment. This calculation was used for both MO_2_ measurements in the respirometers and in the swim-flume. Validation of each MO_2_ value was conducted using R^2^ values from each measure period. MO_2_ measurements with R^2^ values < 0.9 were not used.

**Fig 2:**
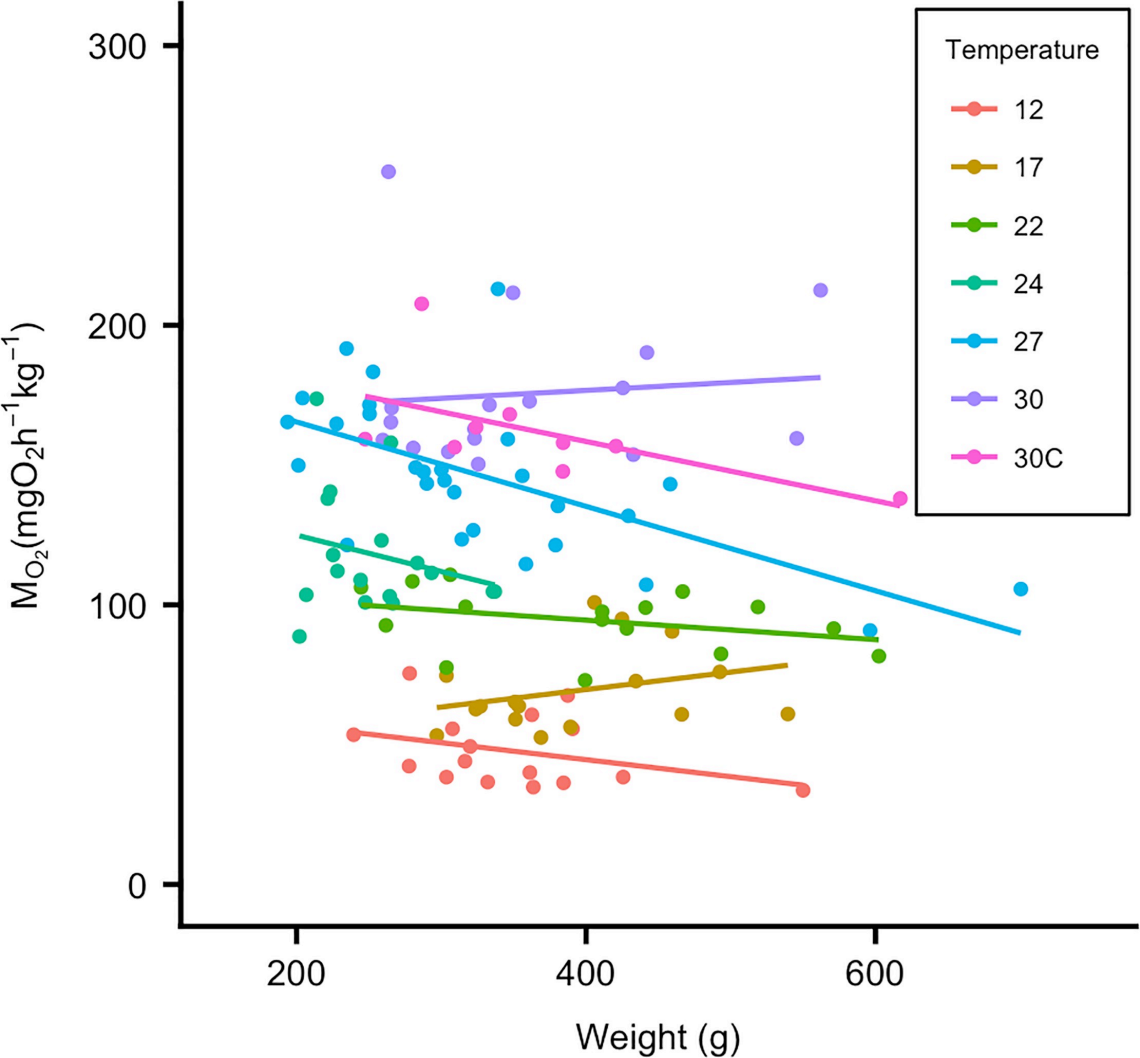
Temperature and body weight both affect standard metabolic rate in black sea bass. SMR (n = 121) for each temperature treatment is plotted against body weight (g). A fitted regression line demonstrates that in addition to the effect of temperature on SMR, body weight also has an effect (*P*<0.05). 30c = 30_chronic_°C treatment.

Standard metabolic rate was calculated from a truncated dataset excluding the hours of elevated MO_2_ values following exercise and by using the 20^th^ quantile of the SMR data in the *calcSMR* package in R [59]. All fish SMR was measured for at least 15 hours in the truncated datasets. Briefly, a frequency distribution of MO_2_ values from the truncated data set was created and the value at the 20^th^ quantile was taken as SMR. The use of the 20^th^ quantile over other methods (i.e. lowest 10%, average of lowest 10 values) is preferred because MO_2_ values naturally fluctuate above and below SMR and avoids potential underestimations of SMR [59]. MMR in the chase and swim-flume protocols was defined as the highest MO_2_ measurement recorded during the respective trials. The difference between MMR methods was analyzed using a Welch two sample *t*-test because sample sizes were unequal. Aerobic scope was calculated from both MMR methods in absolute (AAS = MMR-SMR) and factorial terms (FAS = MMR/SMR). In 2016, fish testing was restricted to three temperatures (24, 27, and 30°C) due to difficulties in maintaining temperatures, and some individuals were tested at more than one temperature due to a limited number of fish obtained for testing. Only fish that were used once were ultimately included in 2016 data analysis to maintain data independence. There was a significant effect of mass on MO_2_ (F_1,117_ = 4.651; *P* < 0.05; Fig 2). Therefore, the effect of temperature on MO_2_ was analyzed using a one-way ANCOVA with weight as a covariate. A Tukey’s HSD *post hoc* test was used to determine significant pair-wise comparisons between temperatures. MO_2_ was adjusted to the mean fish weight (346.9g) using the estimated marginal means from the ANCOVA. The estimated marginal means provides weight-adjusted MO_2_ (MO_2adj_) mean and standard errors for each temperature treatment. These values were used to report results and in graphs where weight had a significant effect on MO_2_. Curves for aerobic scope were modeled using a 3^rd^ degree polynomial fit and were used to estimate a thermal optimum (temperature at the highest AS).

Q_10_ values were calculated for MO_2adj_ between temperature increments, and between the range of temperatures using the formula:

$$Q_{10}={\frac{R_{2}}{R_{1}}}^{10/(T_{2}-T_{1})}$$

where Q_10_ is the temperature coefficient for MO_2_, R_1_ is the MO_2_ at T_1_ and R_2_ is the MO_2_ at T_2_.

S_crit_ was determined by using a broken-stick regression which fits two regression lines through the data: one through the region where MO_2_ remained stable as %O_2_ decreased and one through the portion where MO_2_ decreased linearly with a decrease in %O_2_. The intersection of the two regression lines is the critical point used for S_crit_ [60]. This was analyzed using R code in the *calcO2crit* package from [40]. Because we had a sample size of four fish per temperature treatment, a power analysis was run to determine the statistical power of this small sample size. The effect of weight on S_crit_ was not significant (*P>*0.05) so a one-way ANOVA was used to assess the effect of temperature on S_crit_ and a Tukey’s HSD *post hoc* test was used to determine significant pair-wise comparisons between temperatures. The Metabolic Index (MI) was calculated using the equation from [17]:

$$\Phi =A_{o}B^{n}\frac{PO_{2}}{exp(-\frac{E_{o}}{k_{B}T})}$$

where Φ is the Metabolic Index, *A*_*o*_ is the ratio of rate coefficients, *B*^*n*^ is the body mass scaling, *PO*_*2*_ is ambient O_2_ pressure, *E*_*o*_ is the temperature dependence of baseline metabolic rate, *k*_*B*_ is the Boltzmann’s constant, and *T* is temperature. Here, the S_crit_ data from each temperature treatment is used to determine the *E*_*o*_ and *A*_*o*_ parameters for the equation.

All statistical analyses were performed in R 3.4.3 [61]. Data were checked for assumptions of normality by the visual Q-Q norm plot and statistically with the Shapiro-Wilk test where *P* > 0.05 indicate normally distributed data. Homogeneity was assessed using the Levene’s test where a *P* > 0.05 indicates homogeneity. Data that did not fit assumptions of normality were log-transformed prior to further statistical analysis. Data are presented as mean ± SE and results from statistical analyses are defined as significant at *P* < 0.05.

## Results

### Metabolic rates and aerobic scope

**Fig 3:**
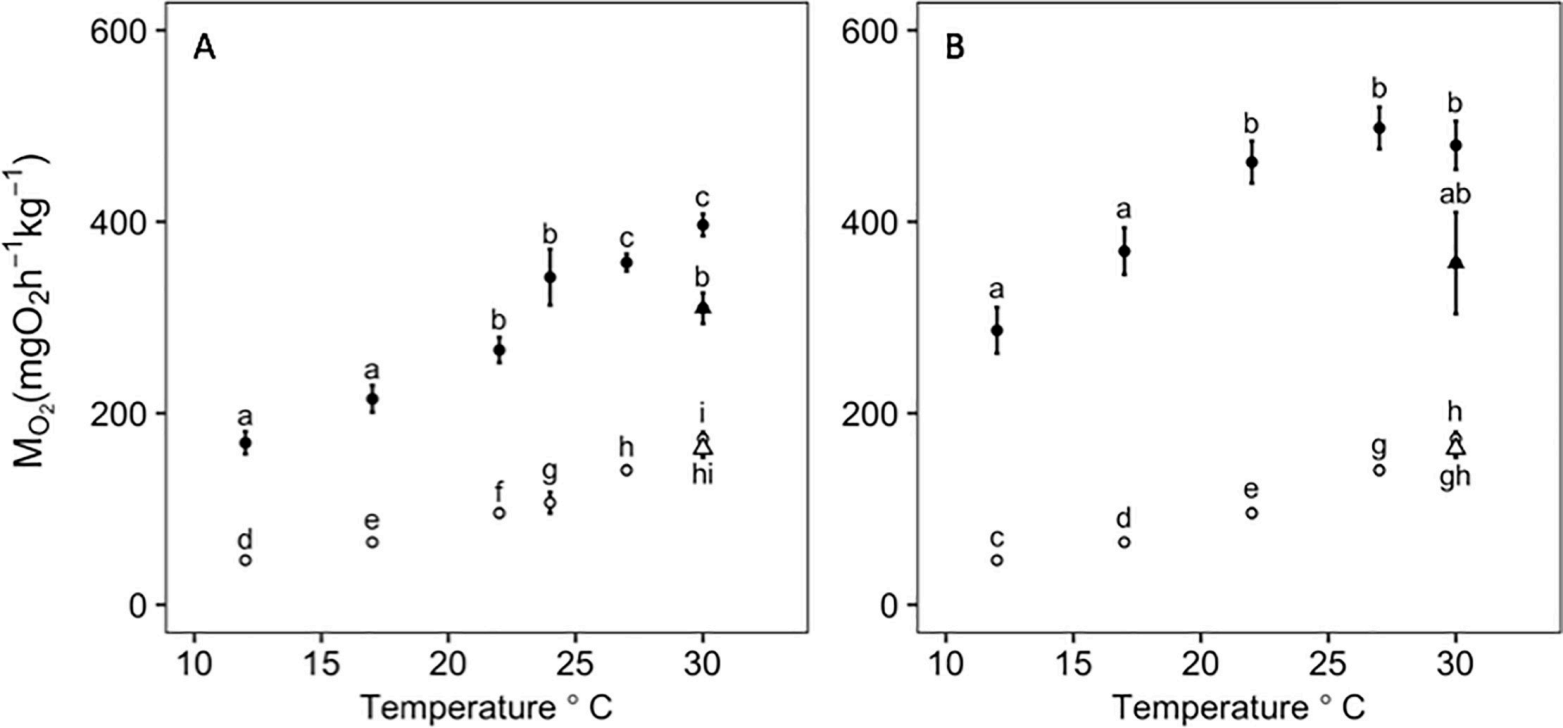
**Effect of temperature on standard metabolic rate and maximum metabolic rate measured with a chase and a swim-flume method.** MMR (solid circles) and SMR (open circles) presented as mean ± s.e. normalized to a mean weight of 346.9g for each temperature treatment for chase method MMR (A) and swim-flume method MMR (B). SMR is slightly different between (A) and (B) based on which fish were used for the respective MMR method. The 30_chronic_°C group is denoted by triangles. Tukey *post hoc* significance between treatments is shown by letters where data points with different letters indicate a significant difference (*P*<0.05).

SMR increased significantly with temperature (Fig 2A and 2B) and there was a significant effect of weight and temperature*weight interaction on SMR (*P* < 0.05; Table 1). SMR values obtained from the 20^th^ quantile were within one standard deviation from the truncated dataset mean for each fish. While the results for the two MMR methods differed significantly (*P <* 0.05 for all temperatures), temperature, weight and temperature*weight interaction all had a significant effect on MMR using either method (*P* < 0.05; Table 1). The chase MMR increased continuously with temperature, while the swim-flume MMR increased with temperature up to ~27°C (Fig 3A & 3B). The MMR values from the swim-flume were consistently higher across the temperature range than from the chase method, indicating that the metabolic rate reached during the chase likely was not the maximum possible for this species.

**Table 1:** ANCOVA results for metabolic rates and aerobic scope.

Variable	Effect	DF	*F*-value	*P*-value^a^
AAS (chase)	Temperature	6, 105	13.877	**< 0.001**
	Weight	1, 105	2.082	> 0.05
	Temperature*weight	6, 105	2.106	0.0586
AAS (flume)	Temperature	5, 48	6.185	**< 0.001**
	Weight	1, 48	6.599	**< 0.05**
	Temperature*weight	5, 48	4.033	**< 0.01**
MMR (chase)	Temperature	6, 105	50.327	**< 0.001**
	Weight	1, 105	9.267	**< 0.01**
	Temperature*weight	6, 105	2.281	**< 0.05**
MMR (flume)	Temperature	5, 48	16.244	**< 0.001**
	Weight	1, 48	8.927	**< 0.01**
	Temperature*weight	5, 48	3.147	**< 0.05**
SMR	Temperature	6, 105	136.613	**< 0.001**
	Weight	1, 105	12.282	**< 0.001**
	Temperature*weight	6, 105	2.489	**< 0.05**

AAS = absolute aerobic scope, MMR = maximum metabolic rate, SMR = standard metabolic rate

^a^*P*-values calculated from ANCOVA; bolded values are significant.

**Fig 4:**
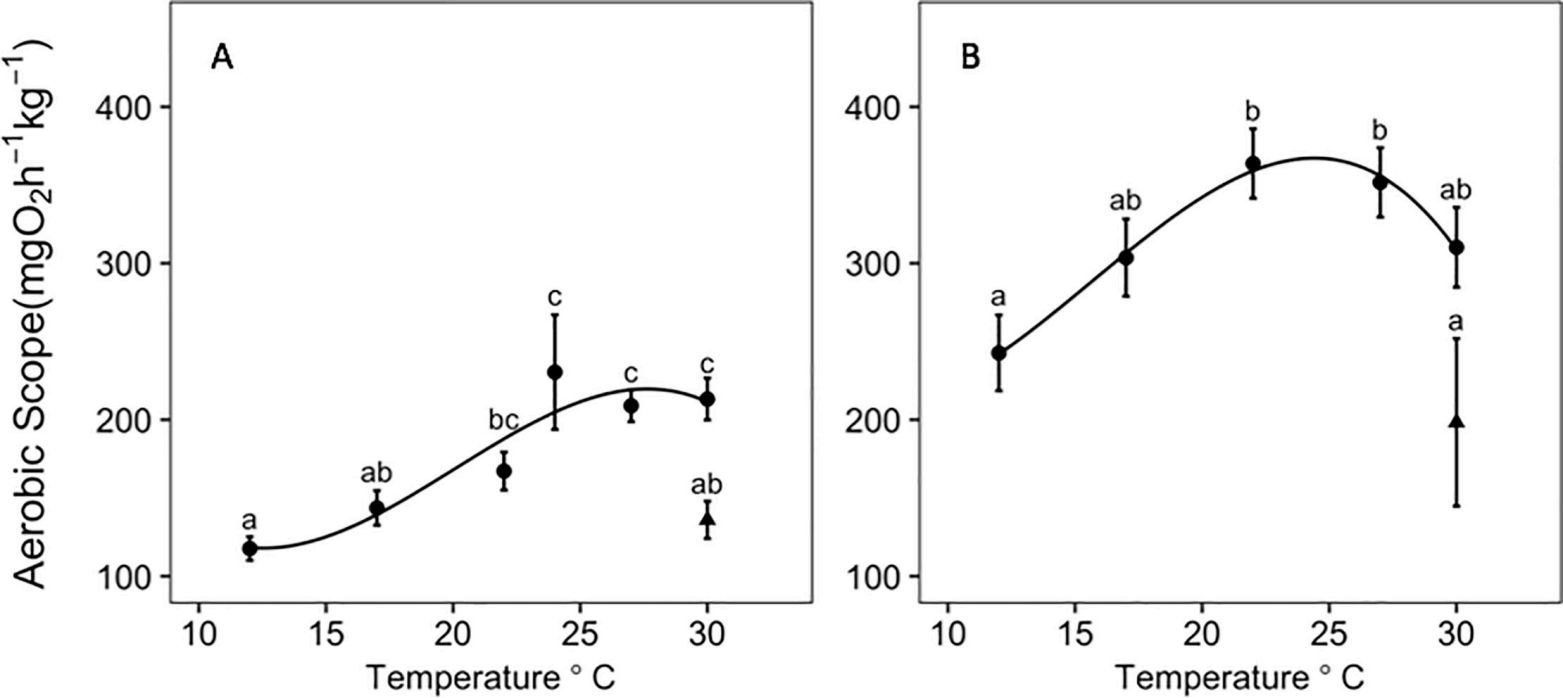
**Effect of temperature on black sea bass aerobic scope.** Aerobic scope (mean ± s.e.) of black sea bass normalized around a mean weight of 346.9g at each temperature treatment with the 30_chronic_°C group denoted by the black triangle. Letters indicate Tukey *post hoc* significance between groups where data points sharing a letter are not significantly different (*P*<0.05). Aerobic scope curves were generated from a) the chase MMR method (*y = 180*.*17 + 89*.*15x – 15*.*40x*^*2*^*–21*.*55x*^*3*^*; R*^*2*^ *= 0*.*878*) and b) swim-flume MMR method (*y = 314*.*36 + 63*.*29x – 68*.*26x*^*2*^*–19*.*65x*^*3*^*; R*^*2*^ *= 0*.*994*).

While the chase method did not achieve MMR, it still provided an estimate of submaximal exercise performance across a temperature range. The MMR achieved using the chase method increased continuously with temperature and reached a maximum adjusted value of 396.65±11.48 mg O_2_ kg^-1^ hr^-1^at 30.0°C (the highest temperature measured; Table 2; Fig 3A). The MMR measured using the swim-flume reached a maximum of 497.96±21.92 mgO_2_ kg^-1^ hr^-1^ at 27°C (Table 2; Fig 3B). The AAS using the swim-flume method reached a maximum, typically referred to as “T_opt_” at ~24.4°C (Fig 3B). There was a significant effect of temperature, weight, and the temperature*weight interaction on AAS (*P* < 0.05) calculated individually from both MMR methods (Table 1). Using different MMR methods resulted in differences in the shape of the AAS curve (Fig 4A & 4B) and the estimated thermal optimum with consequences for its interpretation. The 24°C treatment was slightly overestimated and had larger standard error for AAS and MMR when normalized to a mean fish weight of 346.9g because the average fish weight for this temperature treatment was 253.9g (see S1 Fig for normalized weights at each temperature). However, the 24°C temperature treatment only used the chase method, which we determined did not provide an accurate estimate of MMR and therefore this overestimation does not impact our conclusions. All SMR, MMR and AAS weight-adjusted values, and S_crit_ values, are reported in Table 2. Q_10_ values are reported in Table 3.

**Table 2:** The MO_2adj_ mean ± S.E. values for all metabolic rates, aerobic scope and hypoxia tolerance.

Temperature (°C)	SMR	Chase MMR	Flume MMR	Chase AAS	Flume AAS	S_crit_
12	46.44±1.94	169.12±11.78	286.57±24.02	117.67±7.55	242.67±24.24	19.65
17	65.27±3.27	215.05±14.15	369.27±24.47	143.63±11.07	303.55±24.70	21.33
22	95.69±4.51	266.03±13.32	462.30±22.01	167.18±12.13	363.73±22.21	21.80
24	106.61±11.03	342.06±29.20^a^	NA	230.39±36.65^a^	NA	NA
27	140.55±4.48	357.44±8.99	497.96±21.92	208.96±10.24	351.65±22.12	31.60
30	173.36±7.05	396.65±11.48	479.88±25.29	213.20±13.33	310.20±25.52	37.88
30_chronic_	163.14±9.28	306.62±16.06	356.82±53.05	136.03±11.90	198.33±53.54	38.63

SMR = standard metabolic rate, MMR = maximum metabolic rate, AAS = absolute aerobic scope, S_crit_ = critical %O2 air saturation

^a^Adjusted MO_2_ values for MMR and AAS at 24°C are overestimated due to the average weight of fish in the 24°C group to be smaller than the average weight for all study fish combined.

**Table 3:** Q_10_ values separated between each temperature increment.

	12–17°C	17–22°C	22–24°C	22–27°C	24–27°C	27–30°C	27-30_C_°C
AS_chase_	1.49	1.35	4.97^a^	1.56	0.72^a^	1.07	0.24
AS_flume_	1.56	1.44	NA	0.93	NA	0.66	0.15
MMR_chase_	1.62	1.53	3.51^a^	1.81	1.16^a^	1.41	0.60
MMR_flume_	1.66	1.58	NA	1.16	NA	0.88	0.33
SMR	1.96	2.15	1.72	2.16	2.51	2.01	1.64

AAS = absolute aerobic scope, MMR = maximum metabolic rate, SMR = standard metabolic rate

^a^Slightly overestimated adjusted MO_2_ for the 24°C fish is reflected in calculated Q_10_ values.

### Critical %O_2_ and metabolic index

**Fig 5:**
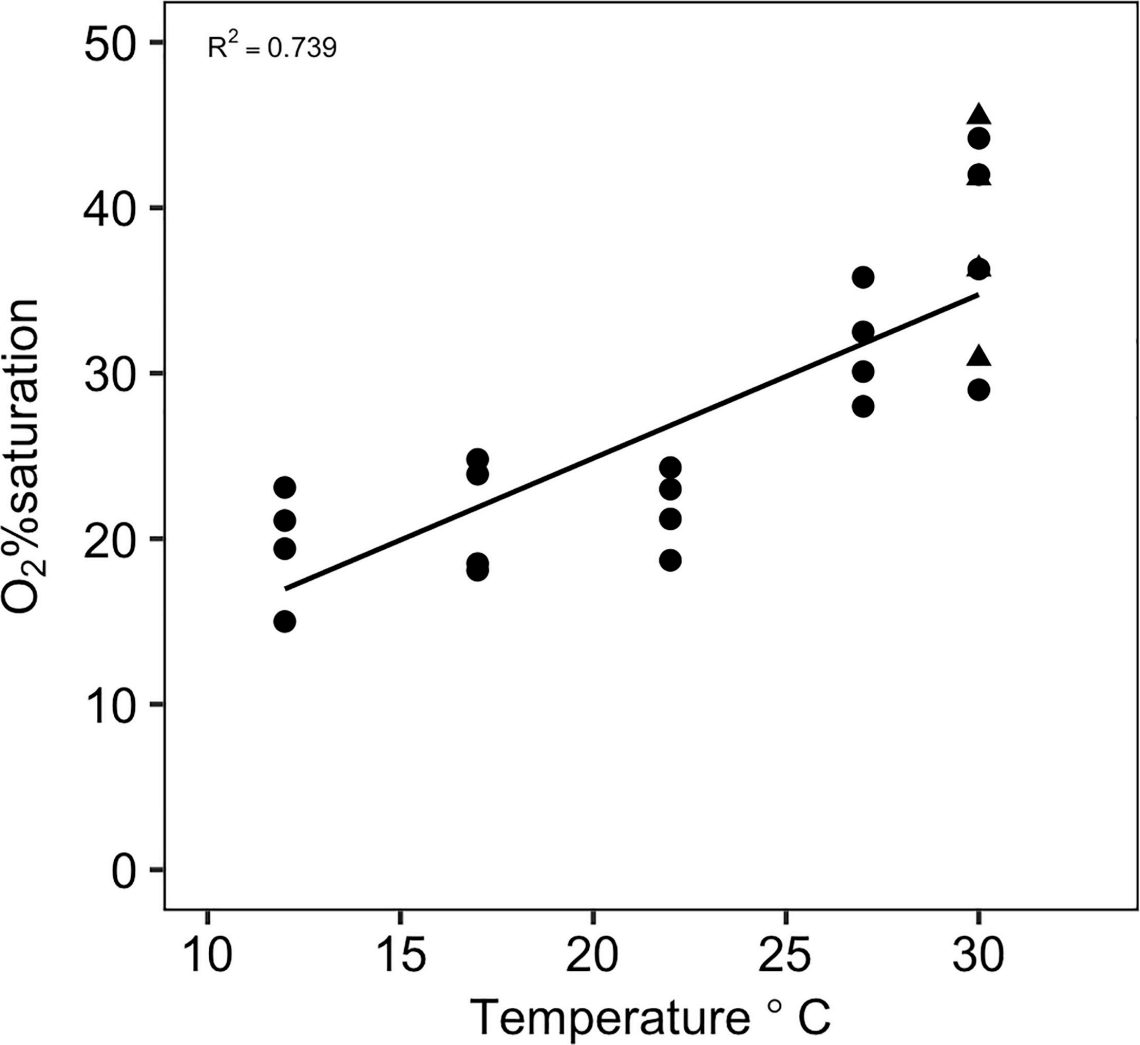
S_crit_ increases with increasing temperature. S_crit_ presented as %O_2_ for each temperature treatment. 30_chronic_°C treatment is denoted by a triangle and there is no significant difference between the 30_chronic_°C and short-term acclimated 30°C treatments. A linear-regression was fitted for these data points (R_2_ = 0.793, *P*<0.001) showing an increase in S_crit_ (e.g. a decrease in hypoxia tolerance) with increasing temperature.

**Fig 6:**
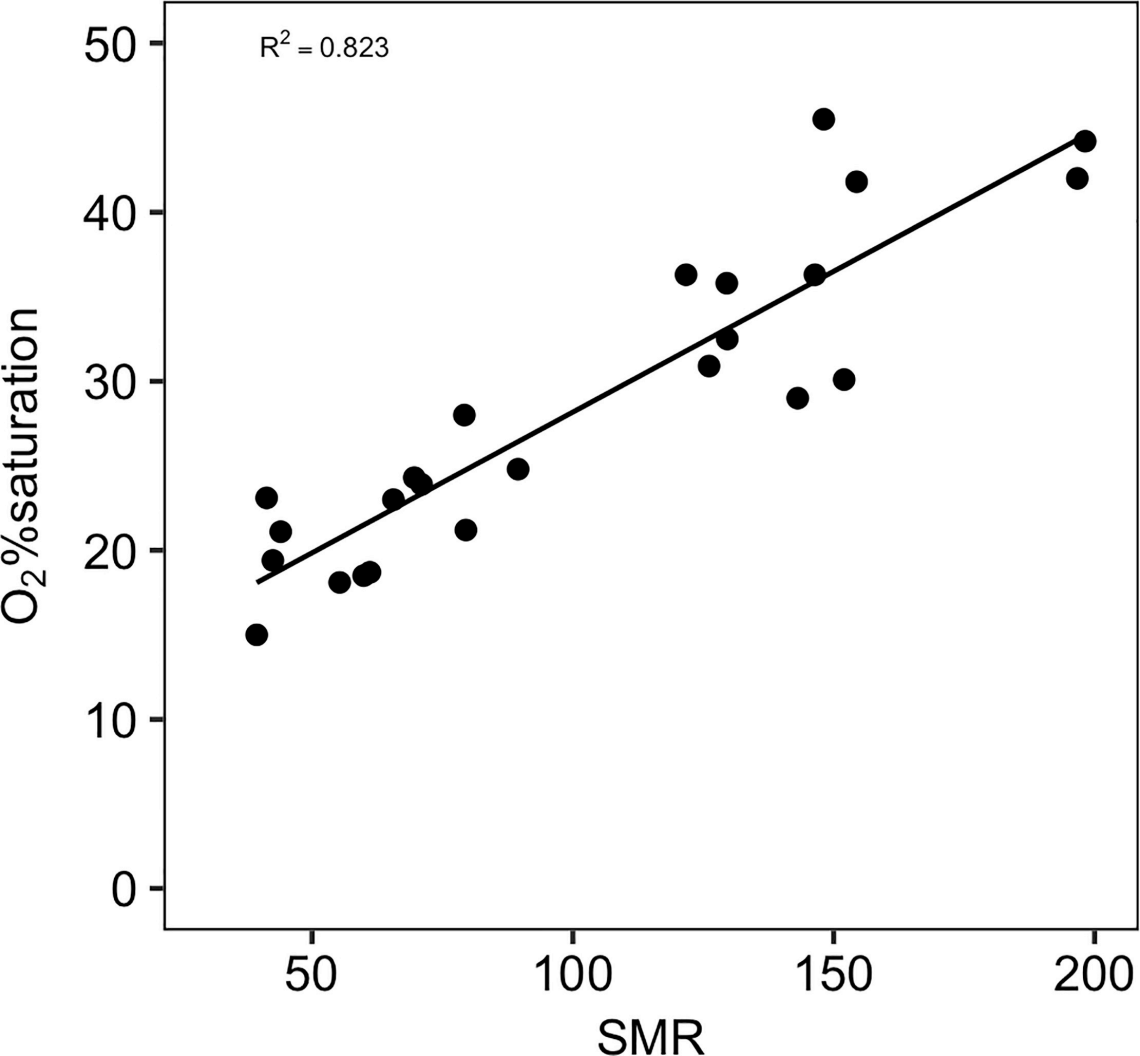
S_crit_ dependence on standard metabolic rate. S_crit_ is plotted against standard metabolic rate measured during the hypoxia experiment. A linear-regression was fitted for these data points (R_2_ = 0.823, *P*<0.001) and shows an increase in S_crit_ as metabolic rates also rise.

**Fig 7:**
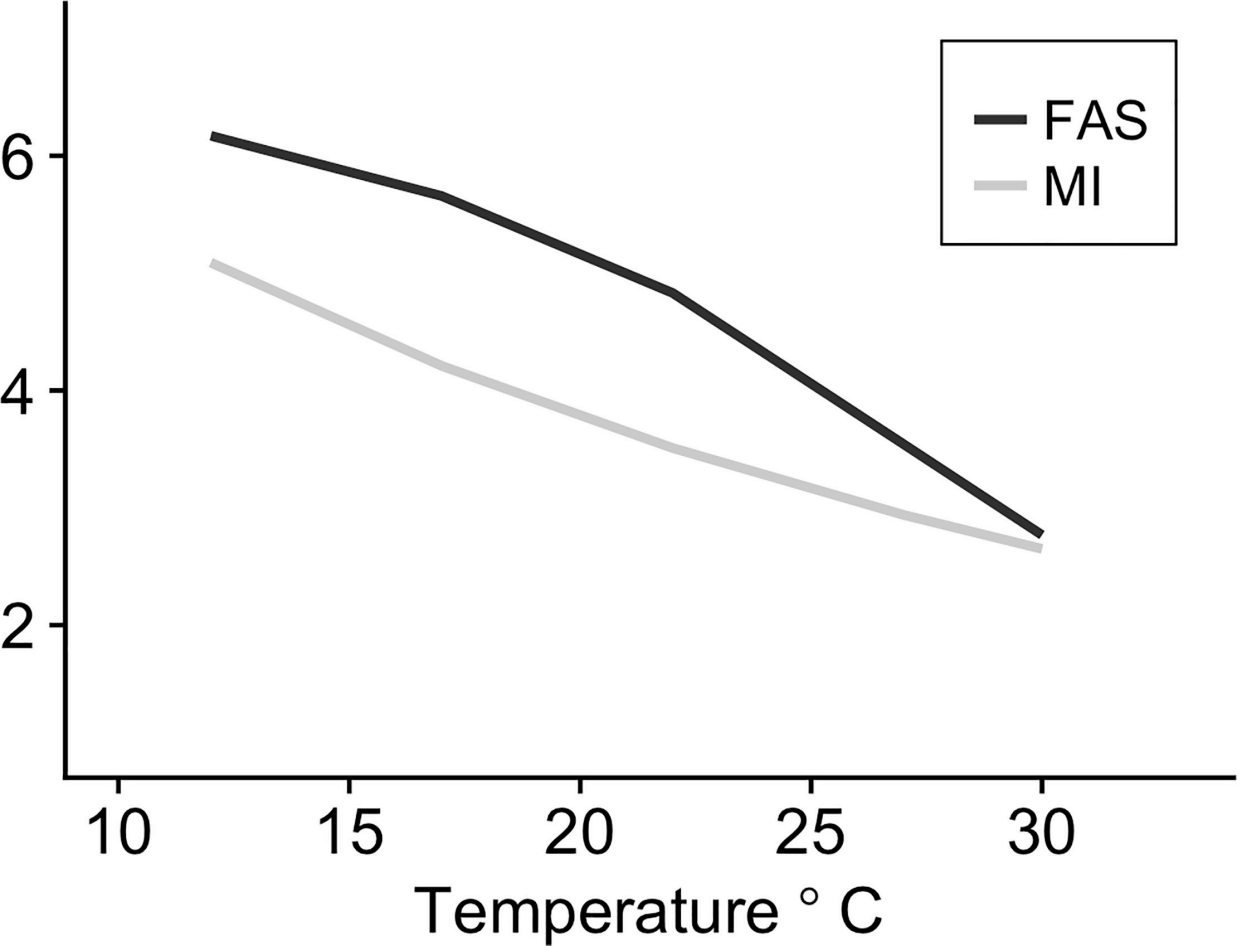
Factorial aerobic scope and metabolic index response to temperature. Factorial aerobic scope (FAS) and metabolic index (MI) plotted against temperature. Trends illustrate a decreasing trend in both measures as temperature increases. Both FAS and MI are unitless measures, but both measures scale similarly.

The power analysis determined that a sample size of four was adequate for statistical testing (*Power* = 1 with *n* = 4, *f* = 1.71 and *sig*. *level* = 0.05). This indicates that there was enough statistical power with a sample size of four fish since the variability between groups was larger than among groups. The critical %O_2_ (S_crit_) increased significantly with increasing temperature (Fig 5; *F*_*5*,*18*_ = 14.023, *P* < 0.05) and significantly increased with SMR (Fig 6; *F*_*1*,*22*_ = 107.6, P < *0*.*001*). There was no significant difference between 12°C (19.65 ± 1.72%O_2_), 17°C (21.325 ± 1.75%O_2_) and 22°C (21.80 ± 1.21%O_2_), but S_crit_ increased significantly at 27°C (31.60 ± 1.67%O_2_) and further at 30°C (37.875 ± 3.39%O_2_). However, non-significance between 12, 17 and 22°C could be due to low sample size. The MI decreased with increasing temperature (Fig 7) but a critical MI (<1) was not observed during this experiment, even under the extreme warm temperatures. A mean critical MI of 3.3 was reported for diverse marine species [17], consistent with the value found near the upper temperature limit (~24.4°C) found here for MMR as well (Fig 7). FAS and MI were of the same magnitude and followed the same decreasing trend with increasing temperatures (Fig 7) supporting the interpretation of MI as another measure of AS.

### Chronic high temperature exposure

The 30_chronic_°C group AAS using both MMR methods significantly decreased when compared to the short-term acclimated 30°C fish that were only held at this temperature for a week (Fig 4). Based on Tukey *post hoc* differences, SMR did not change significantly between the 30_chronic_°C and short-term acclimated 30°C treatments but there was a significant decrease in MMR between the short-term acclimated 30°C and 30_chronic_°C treatments (Fig 3). There was no significant difference in S_crit_ between short-term acclimated 30°C and 30_chronic_°C treatments (Fig 5).

## Discussion

The primary objective of this study was to measure aerobic scope and hypoxia tolerance at a range of ecologically relevant temperatures to assess the potential physiological impacts of ocean warming on and to determine metabolically available habitat for the northern stock of black sea bass. We measured the oxygen consumption rate, a proxy for metabolic rate, following two different exercise protocols. The swim-flume yielded much higher metabolic rates, indicating that the chase method did not elicit MMR. Using the swim-flume MMR, we found that AAS peaked at 24.4°C. S_crit_ increased with increasing temperatures as is typical of most (but not all, [62]) animals, including fishes [57]. The observation that S_crit_ increased with temperature in proportion to SMR, while MMR in the swim-flume did not, suggests that exposure to high temperature did not alter the capacity for oxygen uptake and transport. Chronic exposure to 30°C resulted no change in SMR or S_crit_ but a significant drop in AAS resultant of reductions in MMR (using both methods) implying no loss of oxygen supply capacity as estimated from S_crit_. Instead, this suggests a decrement in muscle function limiting maximum performance with longer exposure to warm temperatures that is not limited by oxygen supply capacity.

Absolute AS typically increases with temperature up to a point, often termed “thermal optima”, and then declines at higher temperatures resulting in a roughly bell-shaped curve. This curve has been identified in fishes that include, but is not limited to, juvenile European sea bass *Dicentrarchus labrax* [63], turbot *Scophthalmus maximus* [64], coho salmon *Oncorhynchus kisutch* [65], and sockeye salmon *Oncorhynchus nerka* [66]. However, some studies have found left- or right-skewed curves (e.g. [27]) while others find that AAS continues to increase up to the critical (lethal) temperature for the species (i.e. no temperature optimum for AAS is identifiable; e.g. [29, 67]). In our study, the black sea bass AAS curve was more bell-shaped with an estimated optimal temperature of 24.4°C. With this finding, the southern portion of the black sea bass range should be considered thermally optimal because bottom temperature is typically 24–26°C during the summer ([46,68]; from U.S. East Coast Regional ESPreSSO model, [69]). However, if the loss in AAS at higher temperatures is due to a failure in muscular performance rather than potential oxygen supply, then 24°C may represent a maximum tolerable temperature rather than a temperature that allows optimal performance. In support of this interpretation, the MI (which closely matches FAS) declines with increasing temperature toward levels (~3 at 27°C in black sea bass) known to limit the geographic range of some species [17]. While the average bottom temperature in the southern portion of the northern stock of black sea bass is near 24°C during the summer months, there has still been a consistent expansion of their range northward into lower temperatures [70] further suggesting that the temperature eliciting maximum AAS is not, in fact, optimal. It is important to note that AAS is only a measured capacity to supply oxygen under maximum sustained exercise [25]. The required scope for other metabolic expenses (i.e. feeding, digestion; [71]) change with temperature in unknown ways and metabolic needs can change seasonally and with ontogeny [54]. Thus, AAS may in this case be an inappropriate predictor of fitness, and does not appear to pinpoint an optimum temperature or correlate with black sea bass distribution. When AAS is measured in the laboratory, important but non-basal energetic requirements (i.e. digestion, reproduction, growth) are removed to provide measurements of SMR. Future studies could benefit from investigating how aerobic capacity changes as other energetic parameters are included in experiments. Finally, the MI, which can be used to predict FAS, can indicate an upper tolerable temperature limit in black sea bass and better explains the northward expansion of these fish. This metric may be more relevant for determining metabolically suitable black sea bass habitat.

Black sea bass in the 30_chronic_°C treatment did not acclimate, indicated by no change in SMR or S_crit_ and a significant decrease in their MMR and AS. Norin et al. [29] similarly found that MMR and AAS in juvenile barramundi decreased significantly following 5 weeks at the highest study temperature (38°C). However, unlike black sea bass in our study, the juvenile barramundi SMR also decreased after the 5-week exposure. This same response has also been found for short-horn sculpin (*Myoxocephalus scorpius*) whose SMR was restored after being held at 16°C for 8 weeks to SMR values that were measured at 10°C [72]. The decrease in SMR can be a compensatory response to high temperatures by reducing energetic costs, but may be accompanied by a reduction in MMR. Importantly, black sea bass in the 30_chronic_°C treatment may have suffered stress from long-term captivity, which could also reduce AAS; time did not permit for a control chronic trial at a cooler temperature (although all fish were held for at least 5 days). Understanding the acclimation potential of black sea bass would benefit from future studies focusing on effects of a chronic treatment at each temperature tested.

S_crit_ increased as temperature increased, most likely caused by rising SMR with higher temperatures, which has been shown in a majority of fish hypoxia studies (e.g. [37]; although see [62]). The 30_chronic_°C group did not have a significant decrease in hypoxia tolerance compared to the short-term acclimation 30°C group, which agrees with no change in SMR between the two 30°C treatments. This suggests that the reduced MMR in 30_chronic_°C fish resulted from reduced muscle function rather than from oxygen supply capacity issues. Black sea bass had lower S_crit_ than striped bass *Morone saxatilis* [73] and summer flounder *Paralichthys dentatus* [33], two important species found throughout the MAB that periodically experience hypoxic water during the summer months. However, when compared with fish that frequently experience hypoxia, such as crucian carp [74], black sea bass were less hypoxia tolerant, especially in warmer water. Along these lines, black sea bass FAS and MI both decreased with increasing temperature (Fig 6). During the summer months when bottom water temperature is warmest along the coastal MAB, periodic hypoxic events occur after large phytoplankton blooms in the surface waters. In the past, these hypoxic events decreased bottom water PO_2_ below ~5.5kPa (26% air-saturation; 2.2 mg L^-1^ at 14°C; [47]), providing a MI of ~1.3 at those temperatures for black sea bass. An MI of 1.3 confers very little aerobic scope for activities beyond basic maintenance costs, and likely does not allow for activities necessary for survival (i.e. foraging, predator evasion) and fitness (i.e. growth, reproduction). Thus, such environments can be tolerated for short periods but are not likely not supportive of a thriving population. At 30°C, even air-saturated water provides a MI of only 2.6 which is near the physiological limits of many species [17]. Therefore, when determining the metabolically suitable habitat, both temperature and oxygen availability must be taken into consideration as both stressors might have synergistic effects on the physiology of this species.

The chase method did not elicit MMR in black sea bass since MMR from the swim-flume method was consistently higher. Which method, chase or swim-flume, provides a more reliable measure of MMR and AAS is debated [23,75]. Whether a maximum rate of oxygen uptake is achieved by either method could depend on the type of swimming the study fish species naturally exhibits in the wild. Norin et al. [29] purposefully used a chase method for juvenile barramundi (*Lates calcarifer*), an ambush predator, that typically swims in quick bursts. In other cases, a fish will exhibit marked post-exercise oxygen consumption (EPOC; [76]), sometimes eliciting MMR minutes to hours after the cessation of exercise [77]. The swim-flume method may be more ecologically relevant for endurance swimming exhibited by pelagic fish such as tunas [75]. Different MMR methods may promote a certain type of swimming which could cause a fish to fatigue before reaching MMR by depleting anaerobic stores, a noteworthy contributor to AAS [78]. For this study, we employed a sprint protocol for the swim-flume, which prompted similar burst swimming as in the chase method. However, during the chase protocol, black sea bass switched almost immediately to burst swimming accompanied with quick turning/flipping movements, compared to a slower transition and continuously straight burst swimming in the swim-flume. The differences in MMR between the two methods could have been related to different swimming types, durations and/or speeds which could recruit more anaerobic resources [79] in the chase method, leading to exhaustion before reaching MMR.

In summary, the results from this study indicate that the northern stock of black sea bass reach a peak in AAS at ~24°C, which is warmer than in the northern portion of their range in the U.S. NES. The MI of 3.8 in air-saturated water, calculated from S_crit_ at 24°C, suggests relatively limited scope for sustained activity at that temperature [17]. We suggest that, rather than an optimal temperature, the peak in MMR and AAS indicates the maximum tolerable temperature, beyond which black sea bass experience a failure in some subcellular or organ systems that contribute to muscle performance. Our study only used individuals from the northern stock that were collected during the summer off of the New Jersey coastline. Metabolic research on the southern stock (south of Cape Hatteras, NC) and/or individuals from the northern stock in waters outside of New Jersey could reveal variation in some of these physiological metrics. However, the distribution of the northern stock of black sea bass has shifted northward [7] and this newly expanded habitat bottom temperature is almost 10°C colder than their apparent thermal optimum for AAS. We believe the preference for cooler waters reflects physiological limitation at higher temperatures, including possible limitation of oxygen supply relative to demand for growth and reproduction (reduced Metabolic Index) despite maintenance of oxygen supply capacity. However, many other factors, including food availability, additional energetic costs (e.g., evading predators, mating), or lower optimal temperatures for other critical processes may be important. This suggests AAS may not be the most appropriate predictor for habitat suitability in this species. Additionally, the northern stock of black sea bass population size has been increasing in the last decade [71], and this increase in biomass could be pushing part of the population northward. Regardless, the chronic exposure experiments presented here suggest little capacity for physiological adjustment to future temperatures. Black sea bass thermal habitat may shrink considerably in the southern region of the MAB as bottom water temperatures reach >27°C and continue to expand into the northern region of the MAB as ocean waters continue to warm, impacting fisheries in these two regions.

## Supporting information

S1 Fig Nomalized fish weights at each temperature treatment. Black sea bass weight (g) normalized to a mean of 0 and standard deviation of 1 for each temperature treatment. The 24°C temperature treatment group only consists of fish collected in 2016, and as seen by almost a difference of one standard deviation, were much smaller than the rest of the experimental fish.
(TIFF)

S1 File Metadata description. (DOCX)

S1 Full Dataset Experimental parameters and raw data. (XLSX)

S1 Table Duration of all intermittent cycles at each temperature. Each intermittent cycle comprises of a flush, wait and measure period (s). The amount of time set at each intermittent cycle component is listed for all temperature treatments.
(DOCX)
