# Correction: The effect of ocean warming on black sea bass (*Centropristis striata*) aerobic scope and hypoxia tolerance

**DOI:** 10.1371/journal.pone.0244002

**Published:** 2020-12-09

**Authors:** Emily Slesinger, Alyssa Andres, Rachael Young, Brad Seibel, Vincent Saba, Beth Phelan, John Rosendale, Daniel Wieczorek, Grace Saba

The ninth author, Grace Saba, is incorrectly noted as the corresponding author. The correct corresponding author is Emily Slesinger. Emily Slesinger's email address is: slesinger@marine.rutgers.edu.
