# Supplementary figures and images for: The effect of ocean warming on black sea bass (Centropristis striata) aerobic scope and hypoxia tolerance
Source: PLoS One. 2019 Jun 13;14(6):e0218390. doi: 10.1371/journal.pone.0218390 (PMC6564031; doi:10.1371/journal.pone.0218390)

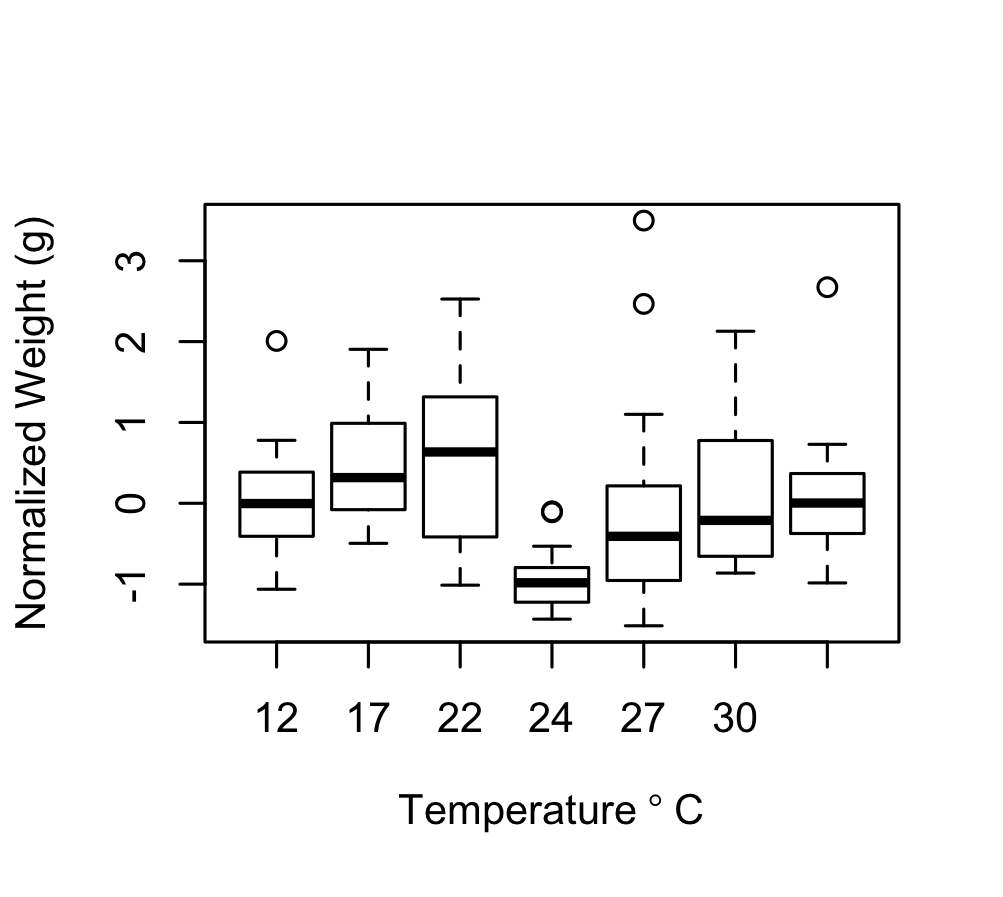

Supplement: S1 Fig — Black sea bass weight (g) normalized to a mean of 0 and standard deviation of 1 for each temperature treatment. The 24°C temperature treatment group only consists of fish collected in 2016, and as seen by almost a difference of one standard deviation, were much smaller than the rest of the experimental fish. (TIFF) [file pone.0218390.s001.tiff]
