# Supplementary material for: The effect of ocean warming on black sea bass (Centropristis striata) aerobic scope and hypoxia tolerance
Source: PLoS One. 2019 Jun 13;14(6):e0218390. doi: 10.1371/journal.pone.0218390 (PMC6564031; doi:10.1371/journal.pone.0218390)
Supplement: S1 File — (DOCX) [file pone.0218390.s002.docx]

S1 Dataset Explanation

Name: Fish #; Description: Individual and unique fish identifier for datasheet; Units: unitless; Missing data identifier: none

Name: Date; Description: Date of experimental start; Units: month/day/year; Missing data identifier: none

Name: Fish_ID; Description: Individual ID given to fish during experiment (note: repeated ID’s between 2016 and 2017 trials); Units: unitless; Missing data identifier: none

Name: Pre-experimental Total Length; Description: Total length (rostrum to longest point on caudal fin excluding long tendrils) measured before experiment; Units: mm; Missing data identifier: none

Name: Pre-experimental Weight; Description: Weight measured before experiment; Units: gms; Missing data identifier: none

Name: Post-experimental Weight; Description: Weight measured after experiment; Units: gms; Missing data identifier: ND (not determined) or NA (not applicable)

Name: Sex; Description: Sex of fish identified with macroscopic gonad identification post euthanasia (note: due to low number of fish in 2016, individuals were ran more than once and sex was identified at the end of the entire experiment. We had individuals change sex throughout the experiment and cannot determine sex during the experiment); Units: unitless; Missing data identifier: ND (not determined) or NA (not applicable)

Name: Target Temperature; Description: Target temperature for the given trial; Units: °C; Missing data identifier: none

Name: Experimental Start Temperature; Description: Temperature measured before start of experiment; Units: °C; Missing data identifier: ND (not determined) or NA (not applicable)

Name: Experimental End Temperature; Description: Temperature measured at the end of experiment; Units: °C; Missing data identifier: none

Name: Experimental Start Salinity; Description: Salinity measured before start of experiment: Units: psu; Missing data identifier: ND (not determined) or NA (not applicable)

Name: Experimental End Salinity; Description: Salinity measured at the end of experiment: Units: psu; Missing data identifier: none

Name: Chase Time; Description: Time that fish was chased in chase tank, chase was defined as over when fish did not respond to touch (did not swim away) and could be removed from the water without resistance; Units: min:sec; Missing data identifier: ND (not determined) or NA (not applicable)

Name: Time In Chamber; Description: Time that the fish was placed in metabolic chamber after the chase; Units: time of day (24hr clock); Missing data identifier: none

Name: Time Out of Chamber; Description: Time that the fish was removed from the chamber and experiment ended; Units: time of day (24hr clock); Missing data identifier: none

Name: Second Trial Designation; Description: Designation for fish after the end of the experiment (either flume trials or hypoxia tolerance trials); Units: none; Missing data identifier: ND (not determined) or NA (not applicable)

Name: Used for Data Analysis; Description: Due to limitations in number of fish in 2016, only fish used once were used for data analysis (yes), fish not used in data analysis (no); Units: unitless; Missing data identifier: ND (not determined) or NA (not applicable)

Name: RMR; Description: Resting metabolic rate that has been background respiration corrected and analyzed using the CalcSMR package in *R*, values in italics were not background corrected since these fish are not used in data analysis; Units: mgO_2_hr^-1^kg^-1^; Missing data identifier: ND (not determined) or NA (not applicable)

Name: MMR Chase; Description: Maximum metabolic rate measured immediately post chase and has been background respiration corrected, values in italics were not background corrected since these fish are not used in data analysis; Units: mgO_2_hr^-1^kg^-1^; Missing data identifier: ND (not determined) or NA (not applicable)

Name: MMR Flume; Description: Maximum metabolic rate measured in the swim-flume trials; Units: mgO_2_hr^-1^kg^-1^; Missing data identifier: ND (not determined) or NA (not applicable)

Name: Pre-trial Background Respiration; Description: Background respiration measured before the experiment by measuring respiration in an empty closed chamber for 2 hours; Units: mgO_2_hr^-1^kg^-1^; Missing data identifier: ND (not determined) or NA (not applicable)

Name: Post-trial Background Respiration; Description: Background respiration measured after the experiment by measuring respiration in an empty closed chamber for 2 hours; Units: mgO_2_hr^-1^kg^-1^; Missing data identifier: ND (not determined) or NA (not applicable)

Name: Absolute Aerobic Scope with Chase MMR; Description: Resting metabolic rate subtracted from maximum metabolic rate from chase method; Units: mgO_2_hr^-1^kg^-1^; Missing data identifier: ND (not determined) or NA (not applicable)

Name: Factorial Aerobic Scope with Chase MMR; Description: Maximum metabolic rate from chase method divided by resting metabolic rate; Units: mgO_2_hr^-1^kg^-1^; Missing data identifier: ND (not determined) or NA (not applicable)

Name: Absolute Aerobic Scope with Flume MMR; Description: Resting metabolic rate subtracted from maximum metabolic rate from flume method; Units: mgO_2_hr^-1^kg^-1^; Missing data identifier: ND (not determined) or NA (not applicable)

Name: Factorial Aerobic Scope with Flume MMR; Description: Maximum metabolic rate from flume method divided by resting metabolic rate; Units: mgO_2_hr^-1^kg^-1^; Missing data identifier: ND (not determined) or NA (not applicable)

Name: Pcrit; Description: Critical O_2_% air saturation level measured during hypoxia experiments; Units: %O_2_ air-saturation; Missing data identifier: ND (not determined) or NA (not applicable)
