# Supplementary material for: The effect of ocean warming on black sea bass (Centropristis striata) aerobic scope and hypoxia tolerance
Source: PLoS One. 2019 Jun 13;14(6):e0218390. doi: 10.1371/journal.pone.0218390 (PMC6564031; doi:10.1371/journal.pone.0218390)
Supplement: S1 Table — Each intermittent cycle comprises of a flush, wait and measure period (s). The amount of time set at each intermittent cycle component is listed for all temperature treatments. (DOCX) [file pone.0218390.s004.docx]

| **Temperature (°C)** | **Flush (s)** | **Wait (s)** | **Measure (s)** |
| --- | --- | --- | --- |
| 12 | 360 | 45 | 600 |
| 17 | 270 | 45 | 480 |
| 22 | 360 | 45 | 360 |
| 24 | 600 | 45 | 360 |
| 27 | 360 | 45 | 360 |
| 30 | 360 | 45 | 300 |

**S1 Table. The amount of time (s) for the flush, wait, and measure period for each temperature trial.**
